# Anti-Inflammatory Effects of the Novel Barbiturate Derivative MHY2699 in an MPTP-Induced Mouse Model of Parkinson’s Disease

**DOI:** 10.3390/antiox10111855

**Published:** 2021-11-22

**Authors:** Seulah Lee, Yeon Ji Suh, Yujeong Lee, Seonguk Yang, Dong Geun Hong, Dinakaran Thirumalai, Seung-Cheol Chang, Ki Wung Chung, Young-Suk Jung, Hyung Ryong Moon, Hae Young Chung, Jaewon Lee

**Affiliations:** 1 Department of Pharmacy, College of Pharmacy, Pusan National University, Busan 46241, Korea; leeseulah@pusan.ac.kr (S.L.); duswl9449@naver.com (Y.J.S.); yujeong0713@naver.com (Y.L.); ajy020603@gmail.com (S.Y.); dgni21@naver.com (D.G.H.); kieungc@pusan.ac.kr (K.W.C.); youngjung@pusan.ac.kr (Y.-S.J.); hyjung@pusan.ac.kr (H.Y.C.); 2 Research Institute for Drug Development, Pusan National University, Busan 46241, Korea; mhr108@pusan.ac.kr; 3 Cognitive Science Research Group, Korea Brain Research Institute, Daegu 41062, Korea; 4 Department of Cogno-Mechatronics Engineering, College of Nanoscience and Nanotechnology, Pusan National University, Busan 46241, Korea; dinakaran@pusan.ac.kr (D.T.); s.c.chang@pusan.ac.kr (S.-C.C.); 5 Department of Manufacturing Pharmacy, College of Pharmacy, Pusan National University, Busan 46241, Korea

**Keywords:** Parkinson’s disease, neuroinflammation, MHY2699, barbiturate, MPTP

## Abstract

Parkinson’s disease (PD) is one of the most common neurodegenerative disorders, and is caused by the death of dopamine neurons and neuroinflammation in the striatum and substantia nigra. Furthermore, the inflammatory response in PD is closely related to glial cell activation. This study examined the neuroprotective effects of the barbiturate derivative, MHY2699 [5-(4-hydroxy 3,5-dimethoxybenzyl)-2 thioxodihydropyrimidine-4,6(1H,5H)-dione] in a mouse model of PD. MHY2699 ameliorated MPP⁺-induced astrocyte activation and ROS production in primary astrocytes and inhibited the MPP⁺-induced phosphorylation of MAPK and NF-κB. The anti-inflammatory effects of MHY2699 in protecting neurons were examined in an MPTP-induced mouse model of PD. MHY2699 inhibited MPTP-induced motor dysfunction and prevented dopaminergic neuronal death, suggesting that it attenuated neuroinflammation. Overall, MHY2699 has potential as a neuroprotective treatment for PD.

## 1. Introduction

Parkinson’s disease (PD) is characterized by reduced dopamine levels in the striatum (STR) and the loss of dopaminergic neurons in the substantia nigra (SN). Neuronal damage is a pathological hallmark of PD and other neurodegenerative diseases and is often caused by activation of the brain immune system. Therefore, considerable research has focused on finding therapeutic approaches to regulate neuroinflammation in neurodegenerative diseases [1].

Neuroinflammation plays important roles in the central nervous system (CNS) because it prompts the repair of damaged tissues, supplies nutrients, defends against external pathogens, and maintains homeostasis [2]. Microglia and astrocytes are unique cells in the CNS and are activated by external stimuli, such as pathogens and traumatic injury, to repair brain damage [3]. On the other hand, chronic neuroinflammation contributes to neurodegeneration, which has been well documented to be closely associated with PD and chronic neuroinflammation. For example, chronically activated glial cells and infiltration by peripheral lymphocytes are observed in postmortem PD tissues in 6-hydroxydopamine (6-OHDA) or 1-methyl-4-phenyl-1,2,3,6-tetrahydropyridine (MPTP)-induced mouse models of PD [4,5,6]. Activated microglia and astrocytes contribute to neurodegeneration by releasing inflammatory cytokines, prostaglandins, and reactive oxygen species (ROS) [7,8]. In previous studies, reactive astrocytes were observed in an MPTP-induced PD model. As a result, it was considered that targeting astroglial regulation might have therapeutic benefits in PD [9,10,11,12].

Barbituric acid has a pyrimidine heterocyclic skeleton and provides the basis for various derivatives called barbiturates [13]. Barbiturates have a range of biological activities, such as sedative-hypnotic and anesthetic activities, achieved by activating gamma-aminobutyric acid (GABA) [14]. They have also been reported to have antioxidant and anti-inflammatory effects in T lymphocytes and in a carbon tetrachloride-induced mouse model of liver fibrosis [15,16,17]. Many barbiturate derivatives have been synthesized to develop pharmacologically profitable agents. On the other hand, the anti-inflammatory effects of barbiturate derivatives have not been studied in the backgrounds of neuroinflammation or neurodegenerative disease.

5-(4-Hydroxy-3,5-dimethoxybenzyl)-2-thioxodihydropyrimidine-4,6(1H,5H)-dione (MHY2699) with a thiobarbiturate backbone has antioxidant effects in a lipopolysaccharide (LPS)-induced mouse model of liver inflammation. MHY2699 can reduce the LPS-induced ROS and peroxynitrite levels in RAW264.7 macrophage cells and mouse liver and significantly attenuate inflammation by suppressing PTEN-Akt-mediated NF-κB activation in RAW264.7 cells [18]. Thus, reports suggest MHY2699 might have antioxidative and anti-inflammatory effects and could be a potential treatment for inflammatory diseases [18,19,20]. This study examined whether the antioxidative and anti-inflammatory effects of MHY2699 are a background of PD. Hence, this study investigated whether MHY2699 has neuroprotective effects in primary cultured astrocytes and MPTP-induced PD mouse models.

## 2. Materials and Methods

### 2.1. Reagents

1-Methyl-4-phenylpyridium (MPP⁺) and 1-methyl-4-phenyl-1,2,3,6-tetrahydropyridine (MPTP) were purchased from Sigma-Aldrich (St. Louis, MO, USA). Dichlorofluorescein diacetate (DCF-DA), DAPI (4′,6-diamidino-2-phenylindole), Alexa Floure 488, and Alexa Flour 568 were obtained from Invitrogen (Portland, OR, USA). Western blot detection reagent (ECL solution) was supplied by Advansta Inc. (San Jose, CA, USA). MHY2699 was synthesized by Prof. Hyung Ryong Moon (College of Pharmacy, Pusan National University, Busan, Korea), as described elsewhere [18].

### 2.2. Primary Astrocyte Culture

Primary astrocyte cultures were established using cells from the Sprague Dawley (SD) rat cortex obtained at postnatal day (PND) 1 or 2 (Daehan Biolink Co., Ltd., Chungbuk, South Korea). Briefly, the cortices were dissected and diffused in ice-cold Hanks’ balanced salt solution (HBSS; Welgene, Daegu, South Korea). The cells were treated with 0.25% trypsin for 30 min at room temperature, washed with HBSS, mechanically dissociated, and plated in Dulbecco’s modified Eagle’s medium (nutrient mixture F12 (DMEM/F12) medium (Gibco, Grand Island, NY, USA) containing 10% FBS (Welgene, Daegu, South Korea) and 1% penicillin–streptomycin (Welgene, Daegu, South Korea) on poly-L-lysine-coated (Sigma-Aldrich, St. Louis, MO, USA) plastic culture dishes. Experiments were performed using 14 to 21-day cultures.

### 2.3. Immunocytochemistry

Primary astrocytes were seeded in 35 mm poly-L-lysine-coated plastic culture dishes and pretreated with MHY2699 (10 μM in DMEM medium containing 0.1% DMSO) for 6 h, or antioxidants (Trolox and NAC in DMEM medium containing 0.1% DMSO) for 2 h. The cells were co-treated with MPP⁺ (500 μM) for 24 h. After treatments, the cells were washed with PBS and fixed with 4% paraformaldehyde (PFA) in PBS (pH 7.4) for 15 min at 37 °C. The cells were then blocked with tris buffered saline and then with Triton X-100 and goat serum (TBS-TS) for 30 min and incubated with the anti-glial fibrillary acidic protein (GFAP) antibody (mouse monoclonal; 1:1000; Cell Signaling Technology, Danvers, MA, USA) overnight at 4 °C. The following day, the cells were washed, incubated with anti-mouse IgG labeled with Alexa Fluor 488 (3 μL/mL) for 3 h at room temperature, and then incubated in DAPI solution (1 μg/mL) at 37 °C for 30 min. The images were obtained using a ZEISS LSM800 confocal microscope (Oberkochen, Germany), and the fluorescence intensity was measured using Image J software. Two or three fields of immune-stained cells in a single dish were photographed, and the fluorescence intensity over the full range of images was measured using Image J software, averaged, and considered as *n* = 1. The GFAP fluorescence intensity was divided by the number of DAPI-positive nuclei because the number of cells in each image could affect the fluorescence intensity.

### 2.4. ROS Measurements

Primary astrocytes were seeded in black 96-well plates (5 × 10^5^ cells/mL) and pretreated with MHY2699 at 10 μM in DMEM medium containing 0.1% DMSO for 6 h and then co-treated with MPP⁺ (500 μM) for 24 h. The cells were then incubated in 80 μM DCF-DA for 30 min at 37 °C and washed twice with warm PBS. Changes in fluorescence intensity were measured repeatedly at 10 min intervals using a fluorescence plate reader (GloMax; Promega, Madison, WI, USA).

### 2.5. Western Blot Analysis

Samples were loaded on 10% SDS-polyacrylamide gels for Western blot analysis and then transferred to Immobilon PS^Q^ transfer membranes (Millipore, Bedford, MA, USA). The membranes were immediately incubated in TBS-T (Tris-HCl-based buffer containing 0.2% Tween 20, pH 7.5) containing 5% nonfat milk at room temperature for 30 min. After washing, the membranes were incubated with primary antibodies: GFAP (rabbit polyclonal; 1:1000; Abcam, Cambridge, MA, USA), phospho-extracellular signal-regulated kinase (p-ERK) (mouse; 1:1000, Cell Signaling Technology, Danvers, MA, USA), ERK (rabbit; 1:1000 Cell Signaling, MA, USA), phospho-Jun N-terminal kinase (p-JNK) (mouse; 1:500, Santa Cruz Biotechnology, Santa Cruz, CA, USA), JNK (rabbit; 1:1000, Cell Signaling, MA, USA), p-p65 (rabbit monoclonal; 1:500, Cell Signaling MA, USA), cyclooxygenase-2 (COX-2) (mouse monoclonal; 1:500, Santa Cruz Biotechnology, Santa Cruz, CA, USA), TH (mouse monoclonal; 1:1000; Chemicon, Temecula, CA, USA), and β-actin (mouse monoclonal; 1:0000; Sigma-Aldrich) in TBS-T at 4 °C overnight. The membranes were then washed for 10 min and incubated with secondary monoclonal anti-mouse and anti-rabbit antibody conjugated with horseradish peroxidase (1:10,000; Santa Cruz Biotechnology) in TBS-T buffer at room temperature for 2 h. The membranes were developed by enhanced ECL and photographed using a cooled CCD camera system (ATTO Ez-Capture; Atto Corp., Tokyo, Japan). The fold changes in the relative protein levels were quantified by densitometry using either total forms of MAPK (ERK, JNK) or β-actin as a loading control.

### 2.6. RNA Isolation and Real-Time Polymerase Chain Reaction (Real-Time PCR)

The cells were homogenized with Trizol reagent (Invitrogen; Carlsbad, CA, USA), and chloroform was added and shaken vigorously for 15 min. The aqueous phase was then transferred to fresh tubes, isopropanol was added, incubated for 15 min at 4 °C, and centrifuged for 15 min at 12,000× *g*. The supernatants were removed, and the pellets were washed with 75% ethanol and centrifuged for 5 min at 8000× *g*. The RNA pellets obtained were dried and dissolved in diethylpyrocarbonate water, and the mRNA concentrations were calculated. mRNA was reverse transcribed to cDNA using SuPrimeCript RT Premix (Genetbio Inc.; Daejeon, South Korea). Real-time PCR was performed using SYBR green master mix (BIOLINE; Taunton, MA, USA) and the CFX Connect System (Bio-rad Inc.; Hercules, CA, USA). The primer sequences were as follows: interleukin (IL)-1β (NM_031512.2) (5′-AAA ATG CCT CGT GCT GTC TG-3′ and 5′-CCA CAG GGA TTT TGT CGT TG-3′); IL-6 (NM_012589.2) (5′-TCA TTC TGT CTC GAG CCC AC-3′ and 5′-GAA GTA GGG AAG GCA GTG GC-3′); tumor necrosis factor-α (TNF-α) (NM_012675.3) (5′-ATT GCT CTG TGA GGC GAC TG-3′ and 5′-GGG GCT CTG AGG AGT AGA CG-3′); GFAP (NM_017009.2) (5′-AGA AAA CCG CAT CAC CAT TC-3′ and 5′ GCA CAC CTC ACA TCA CAT CC-3′); GAPDH (NM_017008.4) (5′-AGA CAG CCC CAT CTT GT-3′ and 5′-ACG GTG AGT CTT CTG ACA CC-3′).

### 2.7. Animals and Drug Administration

Six-week-old male C57BL/6N mice (weighing 20–25 g) were purchased from Daehan Biolink Co., Ltd. (Chungbuk, South Korea). The mice were housed randomly at five animals per cage and maintained under controlled conditions (20–23 °C, under a 12 h light/dark cycle) and provided with food and water *ad libitum*. After an acclimatization period of five days, animals were allocated randomly to four groups. In the MPTP group (*n* = 11), in which animals were administered MPTP intraperitoneally (i.p.) five times at 2 h intervals, or to 1 or 10 mg/kg MHY2699 groups (both *n* = 10), in which animals were pretreated with MHY2699 in phosphate-buffered saline (PBS) containing 5% ethanol and 2% Tween-20) at 1 or 10 mg/kg (i.p.) daily for seven days, and then on the following day were administered MPTP, as described for the MPTP group. Ten animals were also allocated to a treatment-naïve group. The animal protocol used was reviewed and approved by the Pusan National University Institutional Animal Care Committee (PNU-IACUC; Approval Number PNU-2019-2398).

### 2.8. Motor Performance Testing

MPTP-induced motor dysfunction was evaluated using a Rota-rod test, as described previously [21]. All mice underwent training trials for three days to ensure they could maintain themselves on the rod for 180 s at a rod speed of 30 rpm. Training sessions were performed using four consecutive runs. All mice were tested using a Rota-rod speed of 30 rpm for 180 s at 2, 6, 24, and 48 h after final MPTP administration.

### 2.9. Tissue Preparation

To perform histology analysis, the mice were anesthetized 72 h after the last MPTP administration with ethyl ether and perfused intracardially with 0.1 M PBS (pH 7.4) containing 0.9% NaCl and then with 0.1 M PBS containing 4% paraformaldehyde. The brains were removed, postfixed, and cryoprotected. The STR was delineated from 1.54 to −0.34 mm, and SN was −2.54 to −3.88 mm from bregma, according to Paxinos Atlas for the mouse brain **[22]**. Tissue preparation was performed as described previously [23]. Briefly, free-floating brain sections were collected in six series with thicknesses of 40 µm.

### 2.10. Diaminobenzidine Immunohistochemistry

Brain sections (40 µm) were incubated with 0.6% H_2_O_2_ in Tris-buffered saline (TBS; pH 7.5) for 30 min, washed three times with TBS for 10 min, treated with TBS/0.1% Triton X-100/3% goat serum (TBS-TS) for 30 min, and incubated with the primary antibody (anti-tyrosine hydroxylase (TH) antibody in TBS-TS at 4 °C overnight. They were then incubated at room temperature with the appropriate biotinylated secondary goat anti-mouse and anti-rabbit IgG antibodies in TBS for 3 h, and then in an avidin-biotin complex (ABC) solution (Vectastain ABC reagent Elite Kit, Vector Laboratories, Burlingame, CA, USA) in TBS at room temperature for 1 h. After developing a diaminobenzidine (DAB) solution, the images were obtained using a Nikon ECLIPSE TE 2000-U microscope (Nikon, Tokyo, Japan). The quantitative results for dopaminergic neurons were obtained as follows. Five to six sections per mouse containing SN were taken, and the numbers of TH positive dopaminergic neurons in the sections were counted and then divided by the section areas. The average number of TH positive neurons in the SNs of five to six sections of each mouse was calculated. For quantitative analysis for TH immunostaining in STR, the intensity of TH expression in STR (five–six sections per mouse) was measured by FluorChem SP (Alpha Innotech, CA, USA).

### 2.11. Double Fluorescence Immunohistochemistry

Brain sections (40 µm) were blocked with TBS-TS for 30 min at room temperature and incubated with the primary antibodies, i.e., anti-GFAP (mouse polyclonal; 1:500, Cell Signaling, MA) and anti-ionized calcium-binding protein (Iba-1) antibody (rabbit polyclonal; 1:500, Wako, Tokyo, Japan) in TBS-TS at 4 °C overnight. The sections were then washed with TBS, incubated with anti-mouse IgG labeled with Alexa Fluor 488 (3 μL/mL) and anti-rabbit IgG labeled with Alexa Flour 568 (3 μL/mL) for 3 h at room temperature. Images were obtained using a ZEISS LSM800 confocal microscope (Oberkochen, Germany). Two or three sections per mouse containing SN or STR were taken, and the fluorescence intensity over the full range of images in both the left and right sides was measured by Image J software, averaged, and considered as *n* = 1.

### 2.12. Statistical Analysis

An analysis of the variance (ANOVA) and t-test were used to determine the significances of inter-group differences, and a post hoc test was performed using Tukey’s multiple comparisons to compare the groups. The analysis was performed using Prism ver. 7.0. (GraphPad Software Inc., San Diego, CA, USA), and *p* values < 0.05 were considered significant.

## 3. Results

### 3.1. MHY2699 Attenuates MPP⁺-Induced Astroglial Activation and Oxidative Stress in Primary Astrocytes

**Figure 1:**
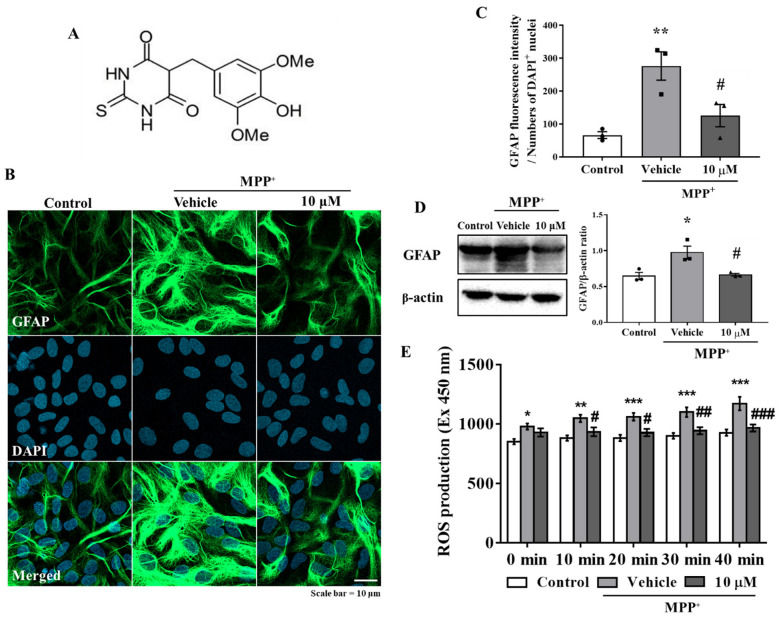
MHY2699 reduced MPP⁺-induced glial activation and ROS levels in primary astrocytes. (**A**) Chemical structure of MHY2699. (**B**) Representative images showing that MHY2699 diminished GFAP fluorescence intensity (an astrocyte marker). The cells were counterstained with DAPI. Scale bar = 10 μm. (**C**) The fluorescence intensity of the GFAP protein was quantified (*n* = 3). (**D**) Western blot analysis confirmed the inhibitory effect of MHY2699 on glial activation. Three independent experiments were performed (*n* = 3). (**E**) ROS levels evaluated using DCF-DA dye showed that MHY2699 suppressed MPP⁺-induced oxidative stress in the primary astrocytes. The values are presented as means ± standard errors (SEM) (*n* = 4). *** *p* < 0.001, ** *p* < 0.01, * *p* < 0.05 vs. naïve control, ### *p* < 0.001, ## *p* < 0.01, # *p* < 0.05 vs. MPP^+^ controls (analyzed by ANOVA with Tukey’s multiple comparisons).

The primary astrocytes were pretreated with MHY2699 for 6 h (10 μM) and then treated with MPP⁺ (final concentration 500 μM) for 24 h. Immunocytochemistry showed that the GFAP (an astrocyte marker) levels were significantly elevated by MPP^+^ and that a pretreatment with MHY2699 reduced these increases (*p* < 0.001, F_2, 6_ = 23.76) (Figure 1B,C). Western blot analysis confirmed that MHY2699 reduced the MPP^+^-induced GFAP expression (*p* < 0.05, F_2, 6_ = 9.104), indicating MHY2699 inhibited MPP^+^-induced glial activation in primary astrocytes (Figure 1D). The levels of ROS production were measured using DCF-DA dye in primary astrocytes to confirm whether MHY2699 has an antioxidative effect. The ROS levels were increased significantly by MPP⁺. MHY2699 suppressed this MPP⁺-induced ROS production (*p* < 0.01, F_8, 45_ = 0.8748) (Figure 1E). These results indicated that MHY2699 has anti-inflammatory and antioxidant effects in primary astrocytes.

### 3.2. Anti-Inflammatory Effect of MHY2699 Was Independent of Its Antioxidant Activity

**Figure 2:**
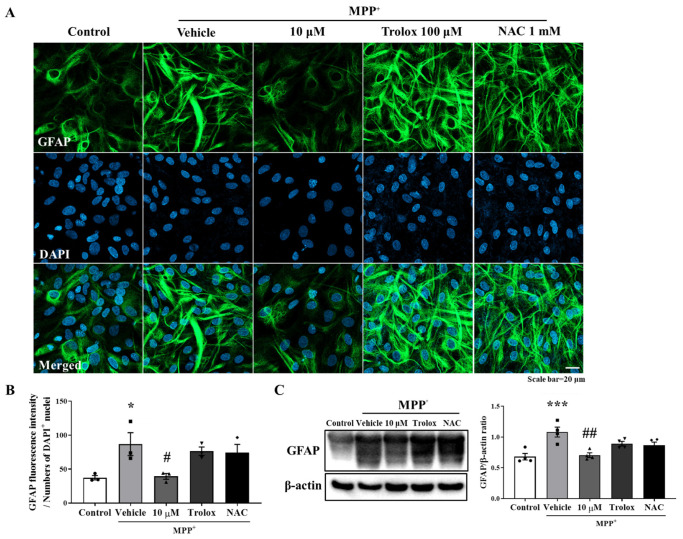
Anti-inflammatory activity of MHY2699 was not related to its antioxidant effect. (**A**) Representative images showing that antioxidants (Trolox and NAC) did not reduce MPP^+^-induced glial activation, which demonstrated that the anti-inflammatory effect of MHY2699 was independent of its antioxidant activity. Scale bar = 20 μm. (**B**) Bar graph of GFAP fluorescence intensity. (*n* = 3). (**C**) Western blot analysis also showed that antioxidants failed to regulate glial activation. (*n* = 4). *** *p* < 0.001, * *p* < 0.05 vs. naïve control, ## *p* < 0.01, # *p* < 0.05 vs. MPP^+^ controls (analyzed by ANOVA with Tukey’s multiple comparisons).

Oxidative stress is a primary risk factor of PD, and antioxidants have been shown to improve the PD symptoms [24]. In addition, oxidative stress causes inflammatory signaling cascades leading to proinflammatory gene expression in glial cells [25]. Therefore, this study investigated whether the anti-inflammatory effects of MHY2699 were mediated by its antioxidant activity. Primary astrocytes were pretreated with 10 μM of MHY2699 for 6 h or well-known antioxidants, 100 μM of Trolox or 1 mM of N-acetyl cysteine (NAC) for 2 h, and activated with 500 μM of MPP⁺ for 24 h. Immunocytochemistry and Western blot analysis showed that Trolox and NAC failed to modulate the MPP^+^-induced glial activation (Figure 2), suggesting that the inhibitory effect of MHY2699 on astroglial activation is a specific pharmacological phenomenon that is unassociated with its antioxidant activity.

### 3.3. MHY2699 Suppressed the MAPK-NF-κB Pathway during Astroglial Activation

**Figure 3:**
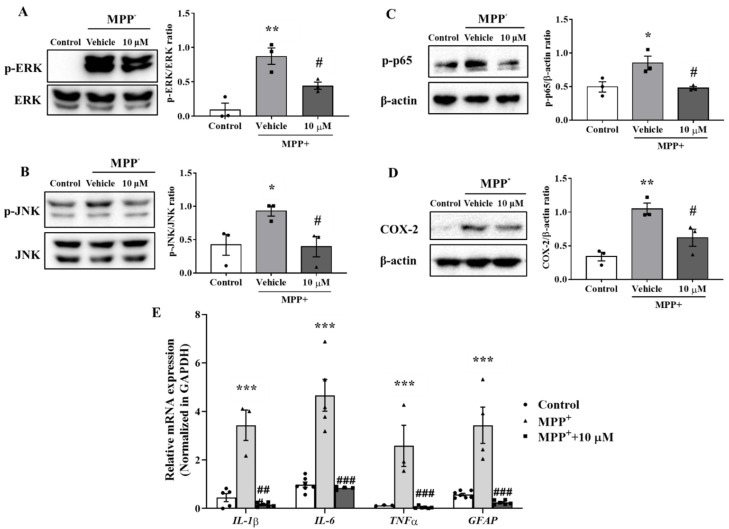
MHY2699 mediated astroglial activation through the MAPK-NF-κB pathway. (**A**,**B**) Western blot analysis showed that MHY2699 repressed MPP⁺-induced ERK and JNK phosphorylation in primary astrocytes (*n* = 3). (**C**,**D**) Western blot analysis showed that MPP⁺ increased the phosphorylation of p65 and COX-2 and that these were decreased significantly by MHY2699, which demonstrated the anti-inflammatory effect of MHY2699-involved regulation of the MAPK-NF-κB pathway. Three independent experiments were performed (*n* = 3). (**E**) Real time-PCR showed that MHY2699 reduced MPP^+^-induced inflammatory cytokines level significantly. The values are reported as the means ± SEs (*n* = 3–7). *** *p* < 0.001, ** *p* < 0.01, * *p* < 0.05 vs. naïve controls and ### *p* < 0.001, ## *p* < 0.01, # *p* < 0.05 vs. MPP^+^ controls (analyzed by ANOVA with Tukey’s multiple comparisons).

Previous studies reported that mitogen-activated protein kinases (MAPKs) such as ERK and JNK are involved in astrocyte activation [26,27]. The mechanism responsible for the anti-inflammatory effect of MHY2699 was examined by pretreating the primary astrocytes with 10 μM MHY2699 for 6 h and co-treating them with 500 μM MPP⁺ for 30 min. Western blot analysis showed that the levels of phosphorylated ERK and JNK were increased significantly by MPP⁺ and that MHY2699 effectively reduced these phosphorylation levels (*p* < 0.05, ERK (F_2, 6_ = 17.68); JNK (*t* = 3.134 for 10 μM)) (Figure 3A,B). Moreover, MPP⁺ increased the phosphorylation of p65 (a subunit of NF-κB) and levels of its downstream inflammatory factor COX-2. On the other hand, MHY2699 effectively suppressed these inflammatory events (*p* < 0.05, p-p65 (F_2, 6_ = 7.816); COX-2 (F_2, 6_ = 13.7)) (Figure 3C,D). Real-time PCR showed that MPP^+^-induced upregulations of inflammatory cytokines (IL-1β, IL-6, and TNF-α) and GFAP in primary astrocytes was significantly suppressed by MHY2699 (Figure 3E; *p* < 0.001, F_6, 47_ = 0.8307). These results suggest that the anti-inflammatory effect of MHY2699 was due to the suppression of astrocyte activation mediated by the MAPK-p65 pathway.

### 3.4. MHY2699 Improved MPTP-Induced Motor Dysfunction in the PD Mouse Model

**Figure 4:**
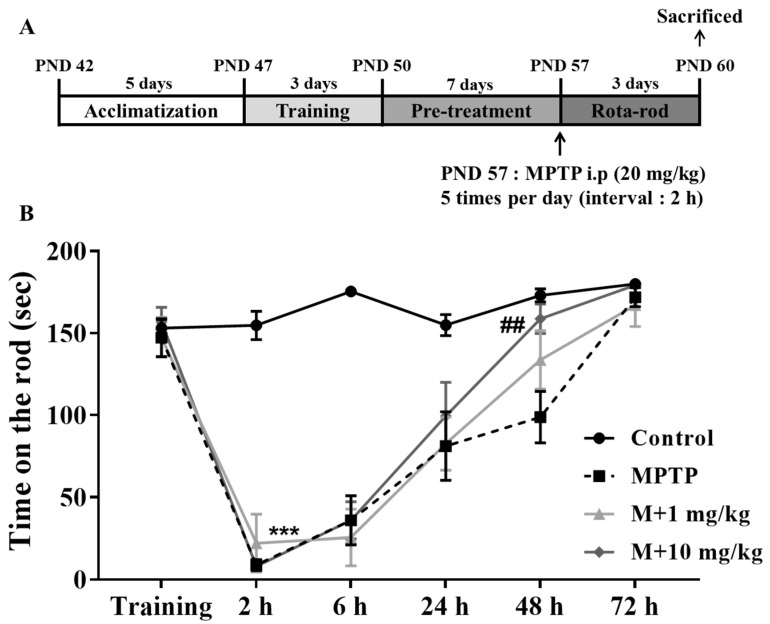
MHY2699 improved MPTP-induced motor deficits in a mouse model of PD. (**A**) In vivo experiment design. (**B**) The Rota-rod test was performed to evaluate motor function. The mice were pretrained for three days to remain on the rod at 15 to 30 rpm. The tests were performed at 2, 6, 24, 48 h, and 72 h after the last MPTP injection at a rod speed of 30 rpm. The values are reported as the means ± SEs (*n* = 10–11 mice/group). *** *p* < 0.001 versus treatment naïve controls and ## *p* < 0.01 versus the MPTP treated group (the analysis was performed using ANOVA with Tukey’s multiple comparisons).

A Rota-rod test, which is commonly used to evaluate the behavioral functions in mouse models of PD, was used to examine the neuroprotective effects of MHY2699. The mice were pretrained for three days to maintain a position on the rod for 180 s at 30 rpm, then pretreated with MHY2699 intraperitoneally at 1 or 10 mg/kg for seven days. The following day, they were administered MPTP (20 mg/kg) five times at 2 h intervals to induce PD-like pathologies (Figure 4A). The MPTP-treated mice showed impaired motor function at 2 h after the final MPTP injection. Interestingly, the motor function recovery was more rapid for the 10 mg/kg MHY2699 pretreated mice than the MPTP-treated mice at 48 h after MPTP administration (*p* < 0.01, F_15, 228_ = 7.09) (Figure 4B). This indicates that MHY2699 could be beneficial to prevent motor dysfunction in PD.

### 3.5. MHY2699 Prevented MPTP-Induced Dopaminergic Neuron Loss in the Nigrostriatal Pathway

**Figure 5:**
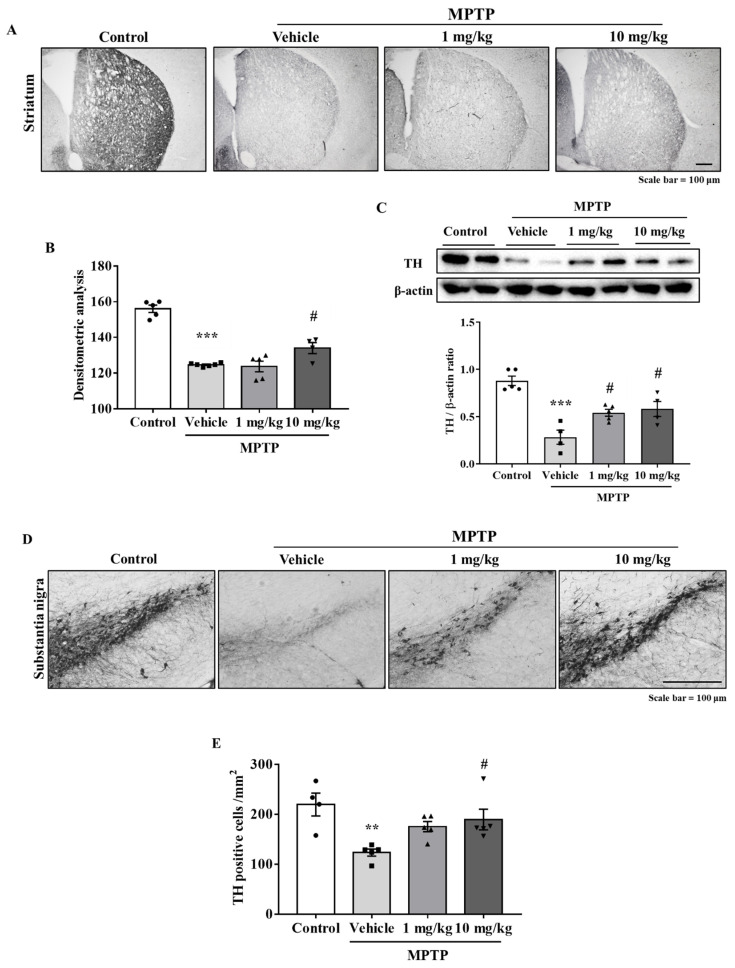
MHY2699 prevented the loss of dopaminergic neurons in the nigrostriatal pathway. (**A**,**B**) Immunohistochemistry was performed to investigate the neuroprotective effects of MHY2699 in the striatum. Scale bar = 100 μm. The values are presented as the means ± SEs (*n* = 4–6 mice/group). (**C**) MHY2699 significantly elevated the TH levels in STR, as determined by Western blotting (*n* = 4–5). (**D**,**E**) TH-positive cell counts showed that 10 mg/kg at MHY2699 prevented MPTP-induced dopaminergic neuronal loss in SN. Scale bar = 100 μm. Values are presented as means ± SEs (*n* = 4–5 mice/group). *** *p* < 0.001, ** *p* < 0.01 vs. the naïve control group, # *p* < 0.05 vs. the MPTP treated group (as determined by ANOVA with Tukey’s multiple comparisons).

Immunohistochemistry was performed using antibodies against tyrosine hydroxylase (TH) (a dopaminergic neuron marker) to determine if MHY2699 protects the dopaminergic neurons from MPTP in the nigrostriatal pathway, including STR and SN. The TH levels were significantly lower in the MPTP-treated group than in the treatment-naïve control group. On the other hand, MHY2699 at 10 mg/kg effectively prevents MPTP-induced TH loss in STR (*p* < 0.05, F_3, 16_ = 49.27) (Figure 5A,B), and this was confirmed by Western blot (*p* < 0.05, F_3, 14_ = 17.28) (Figure 5C). Similarly, in SN, which contains dopaminergic neuron cell bodies, MHY2699 effectively prevented the MPTP-induced loss of dopaminergic neurons (*p* < 0.05, F_3, 15_ = 6.226), (Figure 5D,E). These data suggest the neuroprotective effects of MHY2699 in the nigrostriatal pathway of the mouse PD model.

### 3.6. MHY2699 Suppresses MPTP-Induced Glial Activation in a PD Mouse Model

**Figure 6:**
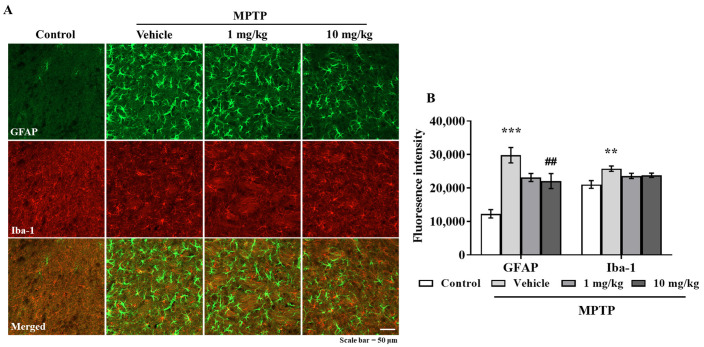

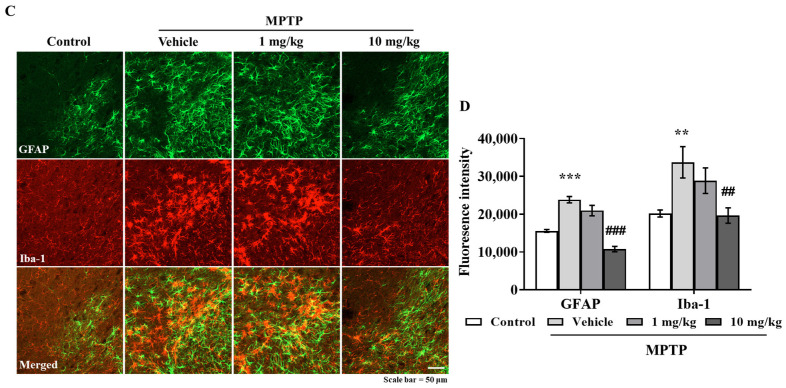
MHY2699 repressed MPTP-induced glial activation in the nigrostriatal pathway. (**A**,**C**) STR (**A**) and SN (**C**) sections were double immunostained using anti-GFAP (green) and anti-Iba-1 (red). Scale bar = 50 μm. (**B**,**D**) Quantitative analyses of GFAP and Iba-1 intensities in the nigrostriatal pathway. The results are presented as means ± SEs (*n* = 3–5 mice/group). *** *p* < 0.001, ** *p* < 0.01, vs. naïve controls, ### *p* < 0.001, ## *p* < 0.01 vs. the MPTP treated group (as determined by ANOVA with Tukey’s multiple comparisons).

Chronic activation of microglia and astrocytes in the nigrostriatal pathway is a characteristic feature of PD. Double immunostaining was performed using GFAP (an astrocyte marker) and Iba-1(a microglial marker) antibodies to determine the effects of MHY2699 on glial activation in PD. Elevated levels of GFAP and Iba-1 were observed in the STR and SN of the MPTP-treated mice, but MHY2699 at 10 mg/kg significantly suppressed these increases in STR (*p* < 0.01, F_3, 31_ = 6.450) (Figure 6A,B) and SN (*p* < 0.01, F_3, 22_ = 0.4049) (Figure 6C,D). These results suggest that the anti-inflammatory effects of MHY2699 might be therapeutically useful in PD.

## 4. Discussion

PD is an age-related neurodegenerative neurological disease [28], and recent studies suggested that the neuroinflammatory responses are responsible for PD pathologies [1]. Glial cells play important roles in neuron protection and maintaining homeostasis in the brain. On the other hand, uncontrolled glial cell activation causes chronic neuroinflammatory responses that trigger the productions of cytokines, chemokines, and oxidative stress [29,30]. From this perspective, chronic neuroinflammation may be a major cause of neuronal dysfunction. Hence, modulating neuroinflammation provides a target for preventing neurodegenerative diseases.

MHY2699 (5-[4-hydroxy3,5-methoxybenzy]-2-thioxodihydropyrimidine-4,6[1H,5H]-dione) was reported to inhibit ROS, peroxynitrite generation, and NF-кB-mediated inflammation [18]. The present study provides the first evidence that MHY2699 might be a useful therapeutic for treating PD symptoms by mitigating the neuroinflammatory responses in neurodegenerative diseases. The anti-inflammatory effects of MHY2699 have been reported in RAW264.7 cells [18], but its ability to inhibit neuroinflammation in primary astrocytes has not been reported. In the present study, MHY2699 also effectively attenuated ROS generation and neuroinflammation triggered by MPP^+^ in primary astrocytes. Experiments with the well-known antioxidants Trolox and NAC were conducted to exclude the possibility that the anti-inflammatory effect of MHY2699 was due to its antioxidant effect (Figure 2). Astrocytes pretreated with these antioxidants remained responsive to MPP^+^ and increased GFAP expression. Indeed, among the MHY compounds (MHY2694~2699) tested, one compound (MHY2698) had an antioxidant effect against MPP^+^ but failed to suppress MPP^+^-induced astroglial activation (data not shown). These results show that the anti-inflammatory effects of MHY2699 were independent of its antioxidant activity, and that MHY2699 has two independent pharmacological properties. Although antioxidants failed to prevent MPP^+^-induced GFAP levels in this study, it was reported that antioxidants could lower neuroinflammation by regulating NRF2 signaling [31]. Therefore, it is plausible that there is still the possibility for the antioxidant to be involved in process of the astrocyte activation by lowering adverse parameters such as proinflammatory cytokines without affecting GFAP levels.

MAPKs such as ERK and JNK, and NF-κB are major regulators of inflammatory reactions. The MAPK pathway activates microglia and astrocytes through the transcription factor, NF-κB, which induces proinflammatory cytokines such as IL-1β, IL-6, and TNF-α, and COX-2 [32,33]. Furthermore, MAPK signaling, including ERK and JNK signaling, plays an important role in the MPP^+^-induced activation of astrocytes [26,27,34]. MHY2699 suppresses astroglial activation through MAPK-mediated NF-κB signaling (Figure 3). A previous study proposed that the anti-inflammatory effect of MHY2699 is mediated, at least in part, by PTEN-Akt-NF-κB signaling in macrophages [18]. LPS binds selectively to Toll-like receptor 4 (TLR4) and activates myeloid differentiation primary response gene 88 (MyD88). MyD88 then induces interleukin 1 receptor-associated kinase (IRAK), which activates MAPKs and the PI3K-Akt signal cascade in macrophages [35,36]. Although the mechanism responsible for the anti-inflammatory effects of MHY2699 probably differs from the LPS-induced inflammatory response in macrophages and MPP^+^-induced inflammatory response in astrocytes, they all involve inhibition of the proinflammatory factor NF-κB.

Neuroinflammation damages the brain and causes glial activation, proinflammatory production, and abnormal neural signaling [37,38]. MHY2699 was administered to the mice, which were treated with MPTP to induce PD pathologies to evaluate the neuroprotective effects of MHY2699, which has both antioxidative and anti-inflammatory effects. MHY2699 effectively mitigated MPTP-induced motor dysfunction, dopaminergic neuronal loss, and glial activation in mouse brains (Figure 4, Figure 5 and Figure 6). When dosed at 10 mg/kg daily, MHY2699 improved motor function significantly and protected the mice from MPTP-induced dopaminergic neuronal loss and neuroinflammation, whereas at 1 mg/kg, it was only effective in suppressing astroglial activation in STR (Figure 6). In previous studies, lichen metabolites such as usnic acid and evernic acid were reported to have a neuroprotective effect in PD mouse model via suppressing astroglial activation [11,12]. Phytochemicals such as silibinin, baicalein, morin, and capsaicin protected dopaminergic neurons by suppressing glial activation in a PD model [26,27,39,40]. Moreover, we have previously reported that PPAR α/γ dual agonist, MHY908 was effective in preventing PD pathologies by its anti-inflammatory effects [10]. Therefore, taken together with current findings, these suggest that regulation of astroglial activation could be beneficial to prevent PD.

In the present study, the anti-inflammatory effect of MHY2699 was examined mainly in primary astrocytes, but its inhibitory effects on microglial activation were not investigated in primary microglia. Double immunostaining analysis showed that MHY2699 effectively suppressed MPTP-induced astroglial activation in both STR and SN in the mouse model (Figure 6). Interestingly, microglial activation was dramatic in SN, and the anti-inflammatory effects of MHY2699 were observed only in SN and not in STR. This was attributed to the time of glial activation, i.e., microglia are activated during the initial stages of neuronal damage, and the astrocytes in the damaged regions mediate the inflammatory response [41,42]. A major dopamine pathway, the nigrostriatal pathway, connects the SN and STR; dopaminergic neuronal cell bodies are located in the SN, and the dopaminergic nerve fibers are located in the STR. Therefore, microglial activation is dramatic in SN when neuronal loss or damage is severe. Hence, MHY2699 might effectively modulate the initial microglial activation during the neurodegeneration process.

Dopamine precursors (L-DOPA), monoamine oxidase-B inhibitors, and catechol-o-methyltransferase inhibitors are used to treat PD. On the other hand, the currently available treatments simply replenish the dopamine loss caused by dopaminergic neuronal losses; they do not address the fundamentals of PD [43]. In addition, the incidence of PD continues to increase, and novel therapies are urgently required to enhance quality of life and reduce the social and financial burdens of this disease. Giving these trends, various methods are undergoing clinical trials. These include stem cell transplantation and trials involving antioxidants, immunotherapy, kinase inhibitors, and neurotrophic factors [44]. Therefore, drugs that modulate the chronic neuroinflammatory response, a major cause of PD, might provide a means of controlling PD.

## 5. Conclusions

Although previous report showed that MHY2699 had antioxidative and anti-inflammatory effects on macrophages and LPS-induced liver damage, the neuroprotective and anti-inflammatory effects of MHY2699 have not been addressed in a PD model. The present study reported, for the first time, that MHY2699 effectively reduced MPP^+^-induced astroglial activation by inhibiting p65 phosphorylation in primary astrocytes. In addition, the study confirmed that MHY2699 ameliorated MPTP-induced motor dysfunction, protected dopaminergic neurons, and suppressed neuroinflammatory responses in PD lesions. In this respect, MHY2699 could offer alternative therapeutic possibilities for targeting neuroinflammation-associated neurodegenerative diseases such as PD.

## Data Availability

Data is contained within the article.
